# Zfp296 Is a Novel, Pluripotent-Specific Reprogramming Factor

**DOI:** 10.1371/journal.pone.0034645

**Published:** 2012-04-02

**Authors:** Gerrit Fischedick, Diana C. Klein, Guangming Wu, Daniel Esch, Susanne Höing, Dong Wook Han, Peter Reinhardt, Kerstin Hergarten, Natalia Tapia, Hans R. Schöler, Jared L. Sterneckert

**Affiliations:** 1 Department of Cell and Developmental Biology, Max Planck Institute for Molecular Biomedicine, Münster, Germany; 2 Department of Stem Cell Biology, School of Medicine, Konkuk University, Seoul, Republic of Korea; 3 University of Münster, Faculty of Medicine, Münster, Germany; University of California, San Diego, United States of America

## Abstract

Expression of the four transcription factors *Oct4*, *Sox2*, *Klf4*, and *c-Myc* (OSKM) is sufficient to reprogram somatic cells into induced pluripotent stem (iPSCs). However, this process is slow and inefficient compared with the fusion of somatic cells with embryonic stem cells (ESCs), indicating that ESCs express additional factors that can enhance the efficiency of reprogramming. We had previously developed a method to detect and isolate early neural induction intermediates during the differentiation of mouse ESCs. Using the gene expression profiles of these intermediates, we identified 23 ESC-specific transcripts and tested each for the ability to enhance iPSC formation. Of the tested factors, *zinc finger protein 296* (*Zfp296*) led to the largest increase in mouse iPSC formation. We confirmed that *Zfp296* was specifically expressed in pluripotent stem cells and germ cells. *Zfp296* in combination with OSKM induced iPSC formation earlier and more efficiently than OSKM alone. Through mouse chimera and teratoma formation, we demonstrated that the resultant iPSCs were pluripotent. We showed that *Zfp296* activates transcription of the *Oct4* gene via the germ cell–specific conserved region 4 (CR4), and when overexpressed in mouse ESCs leads to upregulation of *Nanog* expression and downregulation of the expression of differentiation markers, including *Sox17*, *Eomes*, and *T*, which is consistent with the observation that *Zfp296* enhances the efficiency of reprogramming. In contrast, knockdown of *Zfp296* in ESCs leads to the expression of differentiation markers. Finally, we demonstrated that expression of *Zfp296* in ESCs inhibits, but does not block, differentiation into neural cells.

## Introduction

Pluripotent stem cells have the ability to differentiate into all somatic lineages as well as germ cells, yet they fail to pattern these lineages into a viable embryo. Additionally, pluripotent stem cells can be directed to self-renew by the addition of particular growth factors and can thereby be propagated as immortal cell lines. Therefore, pluripotent stem cells can, in principle, provide a virtually unlimited quantity of a wide variety of specialized cells that can be used for basic research, drug discovery, and regenerative medicine.

Pluripotent stem cells are also capable of reprogramming somatic cells. During gastrulation, somatic cells commit to a particular germ layer. Throughout development, somatic cells continue to differentiate and form various specialized cells. Differentiation can be reversed *in vitro* after the fusion of somatic cells with pluripotent stem cells. The somatic genome is thereby reprogrammed and resembles that of a pluripotent cell [1]. Somatic transcripts are downregulated and silenced, and pluripotent loci are demethylated and transcribed. This reprogramming process is highly efficient and has been observed to occur within one day of fusion [2].

In 2006, Shinya Yamanaka demonstrated that the expression of the four transcription factors *Oct4*, *Sox2*, *Klf4*, and *c-Myc* (OSKM) in somatic cells is sufficient to induce pluripotent stem cell (iPSC) identity [3]. Takahashi *et alia* initially screened expression data for genes specifically expressed in pluripotent stem cells [3]. Screening of 24 candidate genes demonstrated that only four were necessary for inducing reprogramming. Using OSKM, iPSCs have been derived from many different of somatic cell types, including neural stem cells (NSCs) [4], [5], keratinocytes [6], and lymphocytes [7], [8]. iPSCs have been shown to pass every functional test for pluripotency, including germline transmission in chimeras and tetraploid embryo complementation [9], [10]. Using tetraploid embryo complementation, various research groups have obtained mice that were formed entirely from iPSCs [11], [12], [13], [14].

However, the efficiency of iPSC formation induced by OSKM is relatively low. The reprogramming of NSCs induced by OSKM requires approximately 1–2 weeks [4]. In contrast, reprogrammed cells are observed as early as 24 hours after the fusion of NSCs with pluripotent stem cells [2], [15], [16]. This striking difference in efficiency of these two reprogramming methods suggests that pluripotent stem cells express additional factors that can enhance reprogramming.

We sought to identify a novel, pluripotent-specific gene that is capable of enhancing iPSC formation. Using previously obtained expression data, we selected 23 candidate genes and tested each for the ability to enhance reprogramming [17]. Of the tested factors, *zing finger protein 296* (*Zfp296*) led to the largest increase in iPSC formation. We confirmed that *Zfp296* was specifically expressed in pluripotent stem cells and germ cells. *Zfp296* in combination with OSKM induced iPSC formation earlier and with greater frequency than OSKM plus an empty control vector. Through chimera and teratoma formation, we demonstrated that the resultant iPSCs were pluripotent. We showed that *Zfp296* activates transcription of the *Oct4* gene via the germ cell–specific conserved region 4 (CR4), and increases *Nanog* expression when overexpressed in ESCs. We demonstrated that *Zfp296* expression during the differentiation of ESCs inhibits neural induction. Finally, we found that shRNA-mediated knockdown of *Zfp296* in ESCs leads to an increase in the expression of differentiation markers, including *Cdx2*, *Pax6*, and *T*. Therefore, we conclude that Zfp296 is a novel, pluripotent-specific reprogramming factor.

## Results

### OSKM plus *Zfp296* induces reprogramming more efficiently and faster than OSKM alone

We hypothesized that ESCs express pluripotent-specific genes that when added individually to OSKM would enhance the efficiency of reprogramming. Candidate reprogramming genes were drawn from our previously published microarray data [17]. First, we filtered for genes that were expressed specifically in ESCs. These genes were defined as those with at least a four-fold downregulation in expression on day 4 of differentiation and the same or even lower expression on day 7 of differentiation. Candidate genes were required to be expressed in ESCs at a level that was at least four-fold higher than that in NSCs. Finally, candidate genes were selected manually for transcription factors and chromatin regulators, as genes with these functions have previously been shown to enhance iPSC formation. The final list consisted of 23 candidate factors (Table 1).

**Table 1:** Candidate reprogramming factors.

	Expression Relative to ESCs		Clone #^1^	Average #GFP+ colonies^2^
	Day 4	Day 7		
Zfp296	0.23	0.13	I.30936046	97
Esrrb	0.06	0.05	I.40130872	95
Suv39h1	0.12	0.13	I.5352230	90
Tex19	0.04	0.01	n.a./RT-PCR	69
Nr0b1	0.03	0.02	R.C330034L04	61
Rex2	0.03	0.02	I.30462971	58
Tcfcp2l1	0.13	0.12	I.40087602	57
Piwil2	0.13	0.09	I.40131020	46
Gm817	0.10	0.06	R.4932441K12	46
Qkf	0.25	0.26	gift^3^	46
LOC435970	0.02	0.01	R.I1C0022H11	45
Mael	0.11	0.01	I.6744032	42
Prdm14	0.06	0.06	R.**^4^	41
2610305D13Rik	0.20	0.04	R.2610305D13	41
Zhx1	0.21	0.18	I.6406286	36
LOC195331	0.07	0.07	I.30044792	35
Cdyl2	0.06	0.07	I.5690642	32
4933405K07Rik	0.18	0.10	R.4933405K07	32
Mmip2	0.07	0.04	n.a./RT-PCR	26
Grhl3	0.20	0.24	I.3067679	22
Phf17	0.21	0.13	I.5250231	13
Dmrt1	0.10	0.05	I.40129989	12
Rhox6	0.14	0.15	I.30790014	10
empty	n.a.	n.a.	n.a.	41

The 23 candidate reprogramming factors are listed, along with their expression, in differentiated ESCs according to the original array data. Also listed is the cDNA clone used for subcloning into the pMX retroviral expression vectors and the average number of *Oct4-GFP*–positive iPSC colonies obtained after infection of NSCs with the expression vector in combination with OSKM.

1 First initial signifies either IMAGE (I) or Riken (R). Indicated genes were isolated by RT-PCR on ESC RNA.

2 Tested on NSCs in combination with Oct4, Sox2, Klf4 and c-Myc.

3 Qkf cDNA clone was a generous gift from Dr. Tim Thomas.

4 PRDM14 was combined from two Riken clones: 6030400A03 and C330011M19 using an internal EcoRI site.

**Figure 1:**
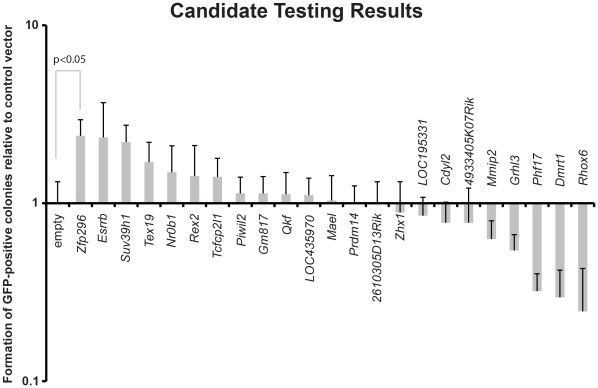
Candidate reprogramming factor testing results. Number of *Oct4-GFP*–positive iPSC colonies formed by NSCs infected with OSKM plus the designated factor relative to OSKM plus an empty retroviral vector. Mean and standard deviations are shown, and *p* values were calculated with t-tests.

Each of these 23 factors was tested individually for the ability to enhance iPSC formation. NSCs with a transgenic *Oct4-GFP* reporter construct were infected with OSKM together with one of the candidate factors. As a control, NSCs were infected with OSKM and an empty retroviral construct (Table 1). The number of green fluorescent protein (GFP)-positive colonies was used as a measure of the reprogramming efficiency and the results are shown in Figure 1. Of our 23 tested factors, a few induced a significant increase in the number of GFP-positive colonies. *Zfp296* and *Esrrb* led to the largest increases in iPSC colony formation. As *Esrrb* has been previously described to enhance iPSC formation, we decided to focus on *Zfp296*. A few factors, such as *Rhox6* and *Dmrt1*, significantly inhibited iPSC colony formation.

**Figure 2:**
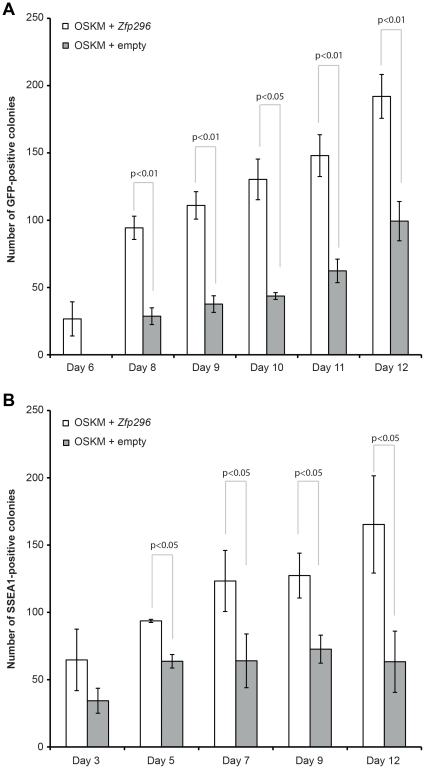
Timing and efficiency of iPSC colony formation. NSCs were infected with either OSKM+*Zfp296* or with OSKM+empty vector. GFP-positive colonies from 30 fields from an automated microscope were counted in three independent replicates for each of the days shown (A). SSEA1-positive colonies from 60 fields from an automated microscope were counted in three independent replicates for each of the days shown (B). Mean and standard deviations are shown, and *P* values were calculated with t-tests.

Next, we sought to evaluate the impact of *Zfp296*, when added to OSKM, on iPSC formation in greater detail. To do this, plates of NSCs infected with either OSKM plus *Zfp296* or OSKM plus empty vector were scanned every day by using an automated microscope, and images were scored manually for the presence of GFP-positive colonies or stage-specific embryonic antigen 1 (SSEA1)-positive colonies. The first GFP-positive colonies were observed on day 6 for NSCs infected with OSKM plus *Zfp296*, in contrast to 8 days for OSKM plus empty vector (Figure 2A). This represents a 25% decrease in the time required for onset of *Oct4-GFP* expression. The number of iPSCs, when scored as either GFP-positive colonies or SSEA1-positive colonies, formed by OSKM plus *Zfp296* was higher compared with that formed by OSKM plus empty control on all time points measured—a significant increase of about 2 fold was consistently observed (Figure 2A and 2B). Therefore, we conclude that *Zfp296* increases the efficiency of OSKM-induced reprogramming of NSCs into iPSCs.

### iPSCs generated with *Zfp296* are pluripotent

**Figure 3:**
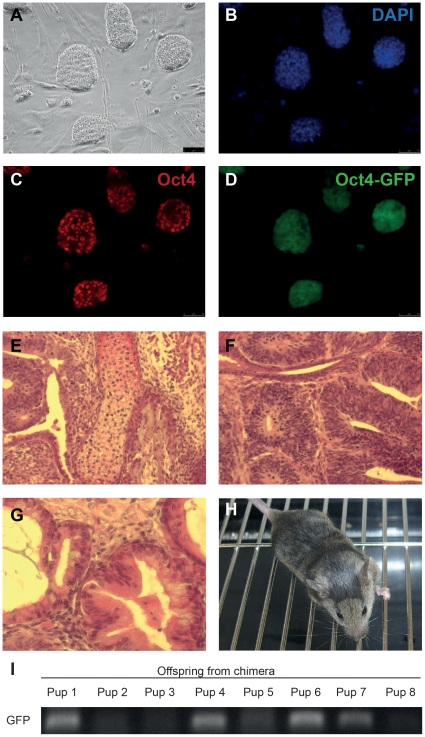
Pluripotency of iPSCs induced with OSKM+*Zfp296*. An iPSC line induced with OSKM+*Zfp296* is shown in phase (A), DAPI (B), Oct4 immunostained (C), or Oct4-GFP (D). Teratomas formed with this iPSC line were composed of cartilage (mesoderm; E), neural rosettes (ectoderm; F), and epithelia (endoderm; G). A chimera formed with this iPSC line is shown (H), and offspring from this chimera carried the GFP transgene, which originated from the iPSCs (I).

**Figure 4:**
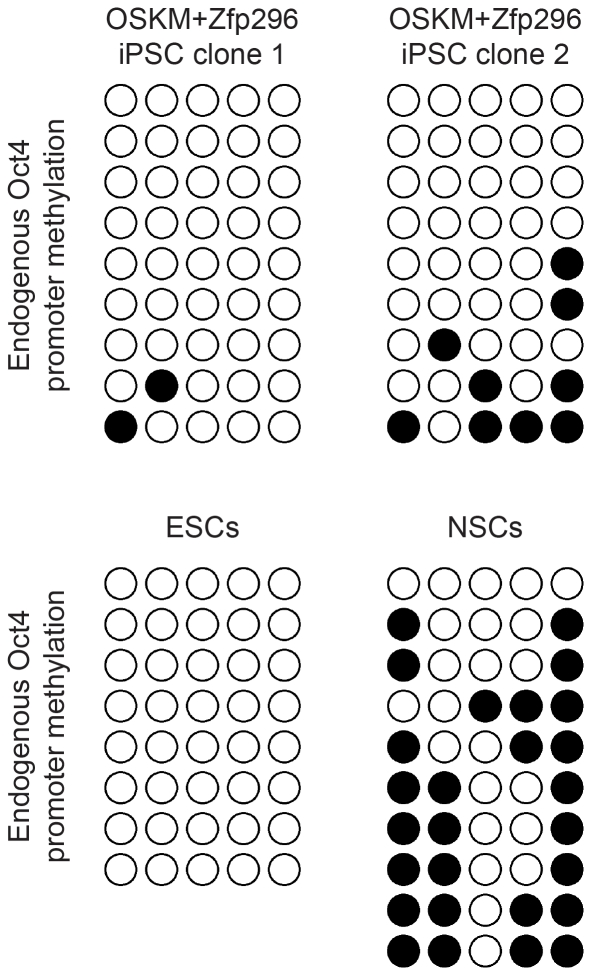
Oct4 promoter methylation. Bisulfite sequencing results assessing the DNA methylation status of the *Oct4* promoter is shown for the indicated cell types. OSKM = *Oct4*, *Sox2*, *Klf4*, and *c-Myc*; ESCs = embryonic stem cells; NSCs = neural stem cells. Open and filled circles represent unmethylated and methylated CpGs, respectively.

The pluripotency of the iPSCs generated by OSKM plus *Zfp296* was then evaluated. To this end, we picked individual iPSC colonies and established individual iPSC lines from NSCs infected with OSKM plus *Zfp296*. Immunostaining confirmed that the iPSCs expressed *Oct4* (Figures 3A–C). The expression of GFP strongly suggests that the endogenous *Oct4* gene had been demethylated and is actively transcribed in the iPSCs generated by OSKM plus *Zfp296* (Figure 3D). Bisulfite sequencing results corroborated our finding that the endogenous *Oct4* promoter had been demethylated in the iPSCs generated by infection of cells with OSKM plus *Zfp296* (Figure 4).

Next, we determined the differentiation potential of the iPSCs using two independent *in vivo* assays. First, iPSCs were injected into immunodeficient mice to form teratomas. Once tumors had formed, they were sectioned and stained. Within individual tumors, forming cartilage (mesoderm), neural progenitors (ectoderm), and epithelial cells (endoderm) were found, indicative of iPSC differentiation into cells with fates from each of the three germ layers (Figures 3E–G). Next, we aggregated the iPSCs with morula-stage mouse embryos and were able to obtain chimeras (Figure 3H). The chimeras were mated, and their offspring were found to carry the *GFP* transgene, which was inherited from the iPSCs, demonstrating germline transmission (Figure 3I). Therefore, we conclude that the iPSCs generated by infection of cells with OSKM plus *Zfp296* are pluripotent, as they are able to differentiate into cells with fates from all three germ layers as well as germ cells.

### *Zfp296* promotes expression of pluripotency markers and inhibits expression of differentiation markers

**Figure 5:**
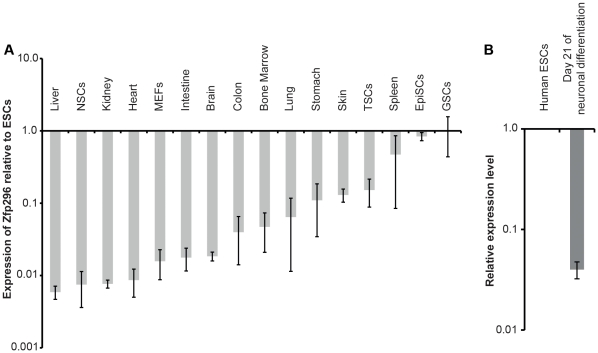
Specificity of *Zfp296* expression. Quantitative real-time RT-PCR results are shown for Zfp296 using RNA from the indicated mouse sources (A) and indicated human ESC-derived cells (B). EpiSCs = epiblast stem cells; ESCs = embryonic stem cells; GSCs = germline stem cells; TSCs = trophoblast stem cells.

We next determined the expression of *Zfp296* in several cell types and organs by quantitative reverse transcriptase–polymerase chain reaction (RT-PCR). *Zfp296* was highly expressed in pluripotent stem cells as well as germline stem (GSCs) (Figure 5A). Significant *Zfp296* expression was also detected in the spleen—but not in the liver, kidney, heart, intestine, brain, colon, bone marrow, lung, stomach, and skin. *Zfp296* was also expressed in human ESCs and was downregulated during ESC differentiation (Figure 5B). This data is similar to that described in a previous report, in which *Zfp296* was found to be primarily expressed by ESCs and testis [18]. However, while Dear and colleagues reported that *Zfp296* expression in the testis was confined to post-mitotic sperm, we demonstrate high levels of *Zfp296* expression in GSCs [18]. Therefore, *Zfp296* is mainly expressed in pluripotent stem cells and cells of the germ cell lineage, including GSCs.

**Figure 6:**
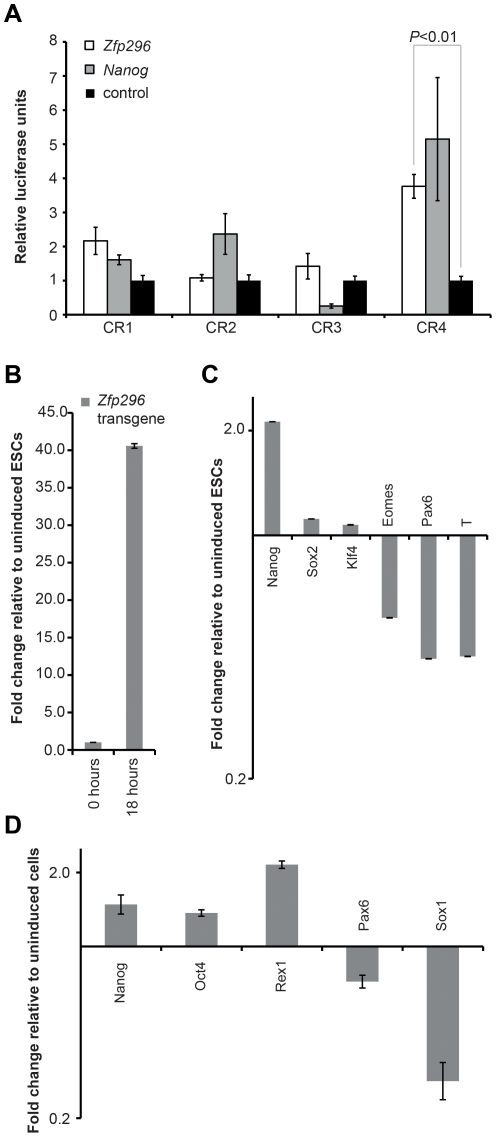
*Zfp296* activates transcription from the *Oct4* promoter. *Zfp296* and *Nanog* were tested by luciferase assays for transactivation of *Oct4* promoter fragments (A). The *P* value for *Zfp296*-induced transcription from CR4 compared with control was less than 0.1, as shown. CR1, CR2, CR3, and CR4 = evolutionary conserved regions 1, 2, 3, and 4, respectively, as defined by Nordhoff *et alia* [20]. ESCs were induced to express *Zfp296* for 18 hours followed by quantitative RT-PCR to assess the expression of transgenic *Zfp296* (B) and indicated marker genes (C) compared with uninduced cells (B). Expression of indicated marker genes on day 7 of neural differentiation in the presence of induced *Zfp296* expression relative to uninduced cells (D).

The ability of *Zfp296* to functionally increase the efficiency of iPSC formation suggests that the factor may interact with the regulatory elements of pluripotent genes. The expression pattern of *Zfp296* was specific to pluripotent stem cells and germ cells, which is similar to the expression pattern of *Oct4*. As such, we hypothesized that *Zfp296* might directly activate transcription via *Oct4* regulatory elements. To test this, we performed a luciferase assay with evolutionary conserved regions of the *Oct4* promoter (Figure 6A). We observed that both *Nanog* and *Zfp296* activated transcription from conserved region 4 of the Oct4 promoter. Of significant interest is the finding that this distal enhancer element is responsible for the expression of *Oct4* in cells of the inner cell mass of pre-implantation embryos, ESCs, and germ cells, all of which also express *Zfp296* [19], [20].

Next, we evaluated the effects of *Zfp296* overexpression in mouse ESCs. To this end, an inducible *Zfp296* vector under the control of tetracycline was introduced into ESCs. 18 hours after induction, transgenic *Zfp296* RNA was upregulated by 40 fold (Figure 6B). Quantitative RT-PCR revealed that at 18 hours, *Nanog* expression was more than 2-fold upregulated (Figure 6C). However, the expression of other pluripotency-associated genes, such as *Sox2* and *Klf4*, was not affected by transgenic *Zfp296*. Prolonged induction of transgenic *Zfp296* led to a morphologic change in the cultured ESC colonies—homogeneously round colonies with a smooth border and surface that indicated improved quality of the ESCs. Consistent with this observation, three markers for differentiation, *Eomes*, *Pax6* and *T*, were all significantly downregulated in the *Zfp296*-induced ESCs. Therefore, we conclude that *Zfp296* directly activates transcription of the *Oct4* gene, and leads to upregulation of *Nanog* expression in ESCs. These results are consistent with the observation that *Zfp296* enhances the efficiency of reprogramming induced by OSKM.

As *Zfp296* overexpression in ESCs led to *Nanog* upregulation, we assessed whether *Zfp296* overexpression would affect the maintenance of ESC pluripotency and inhibit ESC differentiation. The transgenic ESCs were plated under neural induction conditions in the presence or absence of induced *Zfp296* expression (Figure 6D). On day 7 of differentiation, the expression of pluripotency markers *Oct4*, *Sox2*, and *Rex1* was slightly higher and that of the neural differentiation markers *Pax6* and *Sox1* was lower in ESCs overexpressing transgenic *Zfp296* compared with uninduced cells. This result demonstrates that *Zfp296* expression mildly inhibits ESC differentiation, but it is not sufficient to prevent it.

**Figure 7:**
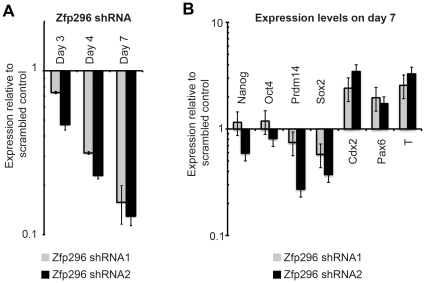
*Zfp296* knockdown in ESCs. Lentiviral shRNA constructs were packaged and used to infect ESCs. Puromycin was added to select for infected cells beginning on day 4. Efficiency of *Zfp296* knockdown is shown on the indicated days after infection (A). Quantitative RT-PCR results are shown for the indicated genes on day 7 after infection (B).

In our final experiment, lentiviral shRNA–mediated knockdown of *Zfp296* was used to determine the role of *Zfp296* in the self-renewal of ESCs. Two constructs with different targeting sequences each resulted in a knockdown of approximately 85% compared with a scrambled control (Figure 7A). Both constructs resulted in upregulation of the differentiation markers *Cdx2*, *Pax6*, and *T* in ESCs compared with the scrambled control (Figure 7B). Although Zfp296 shRNA1 caused downregulation of the expression of the pluripotency markers *Prdm14*, *Rex1*, and *Nanog*, the effects of Zfp296 shRNA2 on pluripotent gene expression were more modest. Expression of the *Oct4* gene was unchanged by reduced *Zfp296* expression. Although our previous luciferase and overexpression results would have predicted a different outcome, it is possible that residual *Zfp296* is sufficient for regulating the CR4 enhancer of Oct4 in ESCs, or that other factors present in ESCs compensate for the lack of *Zfp296*. It is also possible that Zfp296 plays a more significant role in regulating Oct4 expression from CR4 in GSCs. These data show that reduced *Zfp296* expression in ESCs leads to an increase in the expression of markers for differentiation, but this *Zfp296* reduction is not sufficient to prevent ESC self-renewal.

## Discussion

Somatic cell differentiation and fate commitment can be reverted to pluripotency through reprogramming. After fusion of a somatic cell with a pluripotent stem cell, such as an ESC, the somatic cell genome undergoes reprogramming within 24 hours [2]. However, reprogramming mediated by the expression of OSKM requires significantly longer. Therefore, ESCs appear to express additional factors that can enhance the efficiency of reprogramming induced by OSKM. In this study, we sought to identify some of these factors.

We postulated that such additional reprogramming factors would be expressed specifically in ESCs. Using microarray data from ESCs undergoing early neural differentiation, we identified transcripts specifically expressed in undifferentiated ESCs. As all four of the reprogramming genes discovered by Takahashi *et alia* are transcription factors, we focused on transcription factors and chromatin regulators. A final list of 23 ESC-specific genes was selected, and these genes were each cloned into retroviral expression vectors to test for the ability to increase the number of iPSCs formed by OSKM.

Of the 23 genes tested, *Zfp296* led to the largest increase in the number of iPSC colonies formed when combined with OSKM. While the NSCs infected with OSKM expression vectors plus an empty vector consistently formed *Oct4-GFP*–positive colonies on day 8, cells infected with OSKM plus *Zfp296* expression vectors formed significant numbers of *Oct4-GFP*–positive colonies on day 6, with an overall 2-fold increase in iPSC colony formation observed in cultures infected with OSKM plus *Zfp296* compared with those infected with OSKM plus empty vector. Therefore, the addition of *Zfp296* to OSKM induces reprogramming more efficiently and faster than OSKM alone.

We confirmed that the iPSCs formed by OSKM plus Zfp296 expression are pluripotent. First, the iPSCs were found to appropriately express the pluripotent marker *Oct4*. When the iPSCs were injected into immunodeficient mice, teratomas formed containing cells derived from all the three germ layers: ectoderm, mesoderm, and endoderm. Finally, chimeras formed from the iPSCs that had been aggregated with morula-stage embryos. When these chimeras were mated, germline transmission of the *GFP* transgene was detected. Therefore, we conclude that Zfp296 enhances OSKM-induced reprogramming into *bona fide* pluripotent stem cells.

The present study indicates that *Zfp296* is expressed specifically in pluripotent cells and germ cells, and is part of the pluripotent-specific transcriptional network. Previous work has shown that *Zfp296* is expressed in ESCs and post-mitotic sperm [18]. We have extended these results by showing that *Zfp296* is also expressed in pluripotent epiblast stem cells, GSCs, and human ESCs.

The expression pattern of *Zfp296* is similar to that of *Oct4*. Moreover, we have shown that *Zfp296* directly activates transcription of the *Oct4* promoter using the element that is responsible for *Oct4* expression in both ESCs and germ cells—CR4. Overexpression of *Zfp296* in ESCs resulted in increased expression of the pluripotency gene *Nanog* and decreased expression of the differentiation markers *Eomes*, *Sox17*, and *T* relative to uninduced cells. This finding suggests that *Zfp296* plays an important role in both pluripotent stem cells and germ cells. *Zfp296* has been detected at significant levels in the transcriptome of oocytes and 4-cell–stage embryos, suggesting that *Zfp296* may promote the activation of zygotic *Oct4* expression after fertilization [21].

Knockdown experiments in ESCs demonstrated that reduced *Zfp296* levels resulted in an upregulation of differentiation markers. However, we did not observe a consistent reduction in *Nanog* and *Oct4* levels. This was surprising given that *Zfp296* stimulated transcription of CR4 in a luciferase assay and that *Zfp296* overexpression in ESCs increased *Nanog* expression. However, this discrepancy might be explained by residual expression of *Zfp296*. Another possibility is that ESCs express other factors that compensate for the lack of *Zfp296*. It is also possible that *Zfp296* plays a more important role in the regulation of *Oct4* expression in GSCs than ESCs. Therefore, we conclude that *Zfp296* is a novel pluripotent-specific transcription factor that increases the efficiency of OSKM-induced pluripotency and the self-renewal of ESCs.

## Materials and Methods

### Ethics Statement

This study did not involve human participants. All experiments involving animals (e.g. cell transplantation) were carried out in accordance with local institutional guidelines under the protocol 87-51.04.2010.A387, which was approved by Landesamt für Natur, Umwelt und Verbraucherschutz of the state of North Rhine-Westphalia, Germany. *In vitro* experiments were carried out with existing cell lines obtained from previous studies. The appropriate citations are given next to each cell line in the Materials and Methods.

### Microarray data

The OG2 ESCs, which were used to collect the expression data, were derived in the laboratory of Prof. Dr. Hans Schöler, as previously reported [17]. The differentiation, expression data collection, and original analysis of the microarray data have also been described previously [17]. We selected candidate genes based on four main considerations. First, genes had to be either present or marginally expressed in ESCs as defined by the Affymetrix scoring system. Second, genes had to have at least 4-fold downregulation of expression on day 4 of differentiation compared with (undifferentiated) ESCs. Third, genes had to have lower or at most 2-fold higher expression levels on day 7 of differentiation compared with day 4. Fourth, the genes had to have at least 4-fold higher expression in ESCs compared with NSCs. In addition, the final candidates were chosen manually based on their identification as either transcription factors or chromatin factors. A few genes, such as *Piwil2*, were chosen based on a combination of specific expression and known function of potential relevance to reprogramming, despite not being transcription factors or chromatin regulators. Factors that were part of the original screen by Takahashi *et alia* [3] were not considered for selection.

### Candidate reprogramming factor cloning

Whenever possible, cDNA clones were obtained commercially (Table 1). IMAGE clones were preferred and purchased from Open Biosystems. Riken clones were obtained from the FANTOM collection using DNAFORM. The inserts from these original cloning vectors were isolated by restriction digestion and ligated into the pMX vectors [22]. Dr. Tim Thomas kindly provided the Qkf clone. *Tex19* and *Mmip2* were amplified from ESC cDNA using PCR and cloned into pMXs. The ESCs have been described previously, and were derived from preimplantation mouse embryos [17].

The *Zfp296* clone from the IMAGE consortium contained a sequence-verified point mutation (C262T) that resulted in an amino acid change (T88I). This mutation was corrected using site-specific mutagenesis. Primers were designed using the Quikchange primer design webtool (Agilent; https://www.genomics.agilent.com) and used to amplify the corrected version directly from the IMAGE clone. This PCR product was then used to transform *E. coli*, and individual clones were picked and sequenced to confirm correction.

### iPSC formation assay

The NSCs used in this study were derived by the laboratory of Prof. Dr. Hans Schöler and have been reported previously [17]. Retroviral packaging was performed using PlatinumE cells (Cell Biolabs) transfected with a complex of 9 µl of Fugene 6 (Roche Applied Science) and 3 µg of the retroviral plasmid to be packaged. PlatinumE medium consisted of DMEM (Invitrogen), 1× penicillin/streptomycin/glutamine (Invitrogen), and 10% fetal calf serum (FCS; PAA). Two days later, the supernatant was collected and filtered. 50,000 OG2 NSCs were seeded the day before infection on gelatin-coated 6-well plates (Sarstedt) and fed with NSC medium. NSC medium consisted of DMEM/F12 supplemented with N2 supplement, 10 ng/ml epidermal growth factor (EGF), 10 mg/ml basic fibroblast growth factor (bFGF), 50 µg/ml BSA (Fraction V), and 1× penicillin/streptomycin/glutamine (all from Invitrogen). For infection, 100 µl of each of the retroviral supernatants for *Oct4*, *Sox2*, *Klf4*, and *c-Myc* as well as 200 µl of the supernatant for the factor being tested was added to the pre-seeded NSCs. NSC medium was added to a total of 2 ml. Protamine sulfate was added to a final concentration of 6 µg/ml, and the entire mixture was added to the NSCs. After 24 hours, infected cells were washed three times with PBS and fed with fresh NSC medium. Two days later, the cells were again fed with NSC medium. Two days after that, the infected cells were fed with FFES cell medium, and the medium was changed every other day thereafter. FFES medium consisted of Knock-Out DMEM, 16% Knock-Out Serum Replacement (Invitrogen), 4% FCS (PAA), 1% nonessential amino acids (Invitrogen), 1% penicillin/streptomycin/glutamine (Invitrogen), 1% beta-mercaptoethanol (fresh 100× stock solution made by diluting 7 µl of 14.3 M beta-mercaptoethanol [Sigma-Aldrich] in 10 ml PBS [Invitrogen]) and 2,000 units/ml of LIF. Images of GFP-positive colonies were captured on an ArrayScan VTI automated microscope (Thermo) and the number of such colonies were counted manually. 30 images for each day were counted from three independent replicates. For SSEA1, cells infected with the indicated factors in 6-well plates were immunostained and the images captured by the ArrayScan VTI. 60 on each day were counted from three independent replicates.

### iPSC culture

All iPSC lines reported in this study are novel cell lines that were derived from NSCs as described above. These NSCs, as already indicated, were created in the laboratory of Prof. Dr. Hans Schöler and have been reported previously [17]. As these iPSC lines are the derivatives of already established cell lines, neither a new animal protocol nor other approval is necessary. iPSCs were as described above and cultured on irradiated mouse embryonic fibroblasts in ESC medium, which consisted of Knock-Out DMEM (Invitrogen), 20% FCS (PAA), 1% nonessential amino acids (Invitrogen), 1% penicillin/streptomycin/glutamine (Invitrogen), 1% beta-mercaptoethanol (fresh 100× stock solution made by diluting 7 µl of 14.3 M beta-mercaptoethanol [Sigma-Aldrich] in 10 ml PBS [Invitrogen]) and 2,000 units/ml of LIF. Oct4 promoter methylation was analyzed as described previously [23]. The mouse embryonic fibroblasts (MEFs) used in this study were derived in the laboratory of Prof. Dr. Hans Schöler and have been reported previously [17].

### Human ESC culture

The human ESC line HUES6 was purchased from the hESC Collection (Harvard University). The derivation of this line has been reported previously [24]. For differentiation, we used the previously published protocol for motor neuron differentiation by Chambers *et alia* [25].

### Zfp296 Knockdown

Lentiviral shRNA lentiviral knockdown constructs (Sigma: TRCN0000095891 [shRNA1] and TRCN0000095892 [shRNA2]) were produced in 293T cells (ATCC). Cells were transfected with 3 µg of the shRNA construct, 2 µg psPax2 (Addgene 12260), and 1 µg pMD2.G (Addgene 12259) using 18 µl Fugene 6 reagent (Promega). After 3 days, the viruses were concentrated by ultracentrifugation (26,000 rpm, 2 hours, 4°C) and resuspended in 1 ml of DMEM low glucose (PAA). 1×10^4^ ESCs were subsequently infected with 100 µl of concentrated virus, and RNA samples were taken 3, 4, and 7 days after infection. To increase the fraction of infected cells, puromycin selection (1 µg/ml) was started on day 4. As a negative control and for normalization of the real-time PCR analysis, a homemade scrambled shRNA lentivirus was used. For the scrambled knockdown plasmid, the LVTHM plasmid (Addgene 12247) was used as the backbone, with GFP replaced with RFP Tomato [26]. Scrambled shRNA was cloned using ClaI/MluI (both from NEB), with the sequence corresponding to Ito *et alia* 2010 [27].

### Immunostaining

Cells were fixed with paraformaldehyde and permeabilized with Triton X-100. After blocking, cells were stained overnight as indicated. The Oct4 antibody was obtained from Santa Cruz Biotechnology. Secondary antibody staining with AlexaFluor-conjugated antibodies (Invitrogen) was performed the following day. The SSEA1 antibody was obtained from the Developmental Studies Hybridoma Bank (DSHB).

### Real-time RT-PCR

Stem cell marker expression in iPSCs was performed at passages 8 and 10. Total RNA was isolated using the MicroPrep Kit from Zymo Research with on-column DNase I digestion. Reverse transcription was performed using the Multiscribe Reverse Transcriptase Kit (Applied Biosystems). Transcript levels were determined using the ABI PRISM 7900HT Sequence Detection System (Applied Biosystems) and the SYBR green master mix (Bio-Rad). The GSCs used in this study were derived in the laboratory of Prof. Dr. Hans Schöler and have been reported previously [28].

### Aggregation with zona-free embryos

Cells were aggregated and cultured with denuded post-compacted 8-cell–stage mouse embryos as reported with a slight modification [29]. Briefly, 8-cell embryos were flushed from [(C57BL/6×C3H) F1 female mice×CD1 male mice] at 2.5 days post coitum (dpc) and placed in M2 medium (Hogan et al., 1986). Clumps of loosely connected cells (10–20 cells each) with short trypsin treatment were chosen and transferred into microdrops of KSOM medium with 10% FCS under mineral oil; each clump was placed in a depression in the microdrop. Meanwhile, batches of 30–40 embryos were briefly incubated in acidified Tyrode's solution [30] until dissolution of their zona pellucida. A single embryo was place on the clump. All aggregates were assembled in this manner and cultured overnight at 37°C, 5% CO_2_. After 24 hours of culture, the majority of aggregates had formed blastocysts. We transferred 11–14 embryos into each uterine horn of 2.5-dpc pseudopregnant recipients.

### Teratoma analysis

For the teratoma formation assay ∼5×10^6^ cells were injected subcutaneously into the flank of severe combined immunodeficiency (SCID) mice. After 4–5 weeks, the teratoma that had formed was excised, fixed in 4% paraformaldehyde, and subjected to histological examination with hematoxylin and eosin staining.

### Luciferase assay

Transactivation activity was measured using the Dual-Glo Luciferase Assay System (Promega), following the manufacturer's instructions. Briefly, 1×10^5^ 293T cells were plated onto a 24-well plate and transfected with Fugene 6 (Roche). The plasmid DNA consisted of 100 ng of effector DNA (pMX Zfp296, pMX Nanog), 100 ng of pTK-RL (Promega), and 800 ng of pGL3 promoter vector (Promega) containing conserved regions (CR1–4), respectively (Nordhoff et al., 2000). Luciferase activity was measured after 48 hours post-transfection and normalized against the Renilla luciferase activity.

### Induction of transgenic *Zfp296* transcription in ESCs

The overexpression of Zfp296 in ESCs was achieved through an improved Tet-On system previously described by Anastassiadis *et alia* [31]. As a starting point, OG2 ESCs, which carry the OG2_Oct4-GFP transgene, were used. As stated above, these ESCs were derived in the laboratory of Prof. Dr. Hans Schöler and have been reported previously [17]. The transactivator and the tet-responsive Zfp296 transgenes were both sequentially electroporated into the cells by using the Nucleofection II device from Amaxa. The procedure was performed according to the manufacturer's instructions, and subsequent selection was done with puromycin (1 µg/ml; Sigma P8833) for the transactivator and with hygromycin (200 µg/ml; Roth Cp12.2) for the tet-Zfp296 transgene. After a clonal cell line was established, the transgene could be induced by the addition of 2 µg/ml doxycycline (Sigma D9891) and 5×10^−6^ M dexamethasone (Sigma D1756) to the culture medium.
